# Low adverse event rates following voluntary medical male circumcision in a high HIV disease burden public sector prevention programme in South Africa

**DOI:** 10.7448/IAS.17.1.19275

**Published:** 2014-11-17

**Authors:** Rogerio Phili, Quarraisha Abdool-Karim, Oscar Ngesa

**Affiliations:** 1 Department of Public Health Medicine, University of KwaZulu-Natal, Durban, South Africa; 2 Centre for the AIDS Programme of Research in South Africa (CAPRISA), Durban, South Africa; 3 School of Mathematics, Statistics and Computer Science, University of KwaZulu-Natal, Pietermarizburg, South Africa

**Keywords:** medical male circumcision, HIV prevention, adverse events, follow-up visit

## Abstract

**Introduction:**

The provision of voluntary medical male circumcision (VMMC) services was piloted in three public sector facilities in a high HIV disease burden, low circumcision rate province in South Africa to inform policy and operational guidance for scale-up of the service for HIV prevention. We report on adverse events (AEs) experienced by clients following the circumcision procedure.

**Methods:**

Prospective recruitment of HIV-negative males aged 12 and older volunteering to be circumcised at three select public health facilities in KwaZulu-Natal between November 2010 and May 2011. Volunteers underwent standardized medical screening including a physical assessment prior to the surgical procedure being performed. AEs were monitored at three time intervals over a 21-day period post-operatively to determine safety outcomes in this pilot demonstration programme.

**Results:**

A total of 602 volunteers participated in this study. The median age of the volunteers was 22 years (range 12–56). Most participants (75.6%) returned for the 48-hour post-operative visit; 51.0% for day seven visit and 26.1% for the 21st day visit. Participants aged 20–24 were most likely to return. The AE rate was 0.2% intra-operatively. The frequency of moderate AEs was 0.7, 0.3 and 0.6% at 2-, 7- and 21-day visits, respectively. The frequency of severe AEs was 0.4, 0.3 and 0.6% at 2-, 7- and 21-day visits, respectively. Swelling and wound infection were the most common AEs with mean appearance duration of seven days. Clients aged between 35 and 56 years presented with most AEs (3.0%).

**Conclusions:**

VMMC can be delivered safely at resource-limited settings. The intensive three-visit post-operative review practice may be unfeasible due to high attrition rates over time, particularly amongst older men.

## Introduction

The province of KwaZulu-Natal, South Africa, with a HIV prevalence of about 15.8% [1] and low male circumcision rates [2] could potentially benefit enormously from programmatic scale-up of voluntary medical male circumcision (VMMC) services for HIV prevention. Some models have shown that the full coverage of this intervention could avert 2 million new HIV infections and 0.3 million deaths in 10 years [3].

Data from the three VMMC trials that provided the impetus for policy formulation in South Africa demonstrate adverse event (AE) rates that range from 1.5% in Kenya [4], 3.6% in Uganda [5] and 3.8% in South Africa [6]. In industrialized countries where circumcision is typically conducted at birth AE reporting rates range from 0.2 to 0.6% [7]. In resource-limited settings notwithstanding paucity of data and the fact that adult medical male circumcisions are usually undertaken when clinically indicated, AE rates range from 0 to 24% [8–11]. KwaZulu-Natal also has a high burden of other infectious and chronic non-infectious diseases [12]; some of which are contra-indicated for circumcision such as the untreated diabetes leading to some concerns from clinical staff in the province about the safety of VMMC in adults.

Therefore, as part of this pilot demonstration project for provision of VMMC services in public sector facilities we sought to establish the prevalence of co-morbidities, monitor post-operative AE rates and adherence rates to the mandatory post-operative follow-up review visits by circumcised men to inform the programmatic scale-up of MMC services in KwaZulu-Natal.

## Methods

### Project setting and population

This demonstration project was conducted at three public sector health facilities in the province of KwaZulu-Natal; a 24-hour urban community health clinic (CHC), a 874-bed urban district hospital in the Midlands and a 200-bed district hospital in Durban. The Midlands facilities provide services to urban, peri-urban and rural populations and the Durban facility provides services to urban and peri-urban populations. The eligibility criteria for the project were a HIV negative status, an age of 12 years and above, and willingness to return for all mandatory visits for AE assessments. Male volunteers presenting at the study facilities from November 2010 to May 2011 who met these criteria were approached and asked to participate in the study by trained counsellors who referred them to the study staff.

### Procedures

Potential participants above the age of 18 years were asked to sign an informed consent to participate in the study following an intensive counselling on the study goals, risks and benefits of the study by counsellors. Parents and guardians of children under 18 years of age were asked to give informed consent for inclusion of their children. Children aged 12–17 years were required to provide written assent by means of a signature. Consenting volunteers were then provided with information relating to the VMMC procedure, risks of surgery, and the requirements for post-operative wound care by the trained health professionals (nurses).

The participants were provided information on sexually transmitted infections (STIs) symptoms and penile pathology conditions. Pictorial aids were also used to indicate the STIs and penile pathological conditions. Participants were then asked to indicate if they had or had experienced any STI symptoms and other co-morbidities associated with circumcision using a standard questionnaire that was adapted from the WHO sample form for adults and adolescent on the Manual for male circumcision under local anaesthesia [13]. The co-morbidities assessed included genital ulcerative disease (GUD), penile discharge, swelling of the scrotum, pain on micturition, hypertension, blood sugar and bleeding disorders. Penile pathology conditions assessed included phimosis, paraphimosis, epispadias, hypospadias, pain on erection, balanitis and torsion of the penis. Clinical examinations were conducted by trained clinicians (nurses and medical practitioners) to ascertain the self-reported symptoms and to exclude any current STI infections and other co-morbidities. Additional questions on whether participants were on any treatment of comorbidities were also asked to validate the initial responses. Rapid screening using dipstick tests was done for random glucose levels for all participants. Additional questions on whether the participants had undergone HIV testing before presenting for VMMC, reasons for seeking VMMC, and sexual partner status were asked using the standardized questionnaire. Participants with STIs and other treatable conditions were required to complete treatment prior to circumcision. Participants with penile pathology were circumcised using the prescribed alternative methods as described in the WHO Manual for Circumcision Under Local Anaesthesia [13] or referred to urology facilities at appropriate referral hospitals.

All circumcisions were performed by trained medical practitioners in accordance with guidelines provided in the WHO Manual for Male Circumcision Under Local Anaesthesia. All circumcisions were conducted in appropriately equipped on-site theatres.

During the post-surgery recovery period, clients were counselled on wound care, the importance of refraining from coital activity until complete wound healing (estimated to be about six-week post-surgery) and HIV risk reduction strategies. Participants were requested to return for three post-surgical monitoring visits, i.e. day 2, day 7 and day 21 or sooner if any complications occurred prior to these scheduled visit dates.

At follow-up visits, trained health care providers (nurses and medical practitioners) undertook assessments using standardized clinical charts that included monitoring of pain, swelling, haematoma, bleeding, infection, difficulty in urinating, wound disruption/delayed healing, problems with penis appearance, and injury to the glans. Wound healing was confirmed when a healthy scar with no residual exudate or scab formation was evident and after all sutures had been completely absorbed. All AEs were graded by severity as per criteria stipulated in the WHO Manual for Male Circumcision Under Local Anaesthesia as follows:

*Mild*: Evident AEs that resolved without requiring any medical treatment.

*Moderate*: Notable symptoms requiring intervention or treatment by a health care provider or medication (parental, oral or topical).

*Severe*: AEs that led to serious deterioration of patient's health, or resulted in life threatening illness or injury, resulted in permanent injury of a body structure or function, required in-patient hospitalization or prolongation of existing hospitalization or resulted in medical or surgical intervention to prevent permanent impairment to body structure or body function.

Volunteers who returned prior to scheduled dates were seen by the clinicians on the day of presentation and their data were assigned to the nearest scheduled date. Medical treatment was provided to all participants who developed AEs as indicated.

### Data collection and analysis

Data on patient demographics, self-reported symptoms, clinical examinations, and post-surgical follow-up AEs were recorded on patient clinical charts and subsequently captured in a SPSS version 21 database (SAS Institute Inc., Cary, USA). Incomplete charts without information on the circumcision method or with only the patient demographics were excluded in the data analysis. Data analysis was undertaken using the same SPSS system. Bi-variate analysis was undertaken using Pearson's *χ* ^2^. A *p*-value of <0.05 was considered statistically significant. Cases containing a missing value for the variables were not included in the analysis (computation of outcomes based on just the non-missing cases). The primary outcome of the study was the incidence rate of moderate and severe AEs as per standard practice in similar projects [4–6,10]. Pain was not reported in this study as it is often associated with a variety of other conditions.

The study was approved by the Biomedical Research and Ethics Committee of the University of KwaZulu-Natal in November 2010 (Reference No: BF035/010).

## Results

### Socio-demographic characteristics

A total of 775 clients were offered study participation. Fifty seven (57) declined participation, 60 had files with missing information on the circumcision method used, 10 were circumcised with the Tara Klamp, seven had files with only demographic information and 40 had missing files and were excluded from the final sample. A total of 602 HIV negative participants were finally followed up over the 21 days post-circumcision.

The median age of the cohort was 22.0 years (Range: 12–56). Most participants belonged in the 20–24 year-old group (45.2%) and were single males without regular sexual partners (60.6%). The majority of clients (315) were circumcised at the Midlands community health centre (MCHC), 16 at the Midlands district hospital (MDH) and 271 at the Durban district hospital (DDH). Most circumcision procedures were undertaken using the forceps guided method (97.2%; *n*=585) with 2.8%; *n*=17 using the dorsal slit technique. All 17 dorsal slit methods were performed on 16 participants with phimosis and one participant with paraphimosis. Site-stratified data on age, prior HIV testing, coital frequency and relationship characteristics are presented in Table 1. Prior HIV testing was uncommon at all sites (*p*<0.05) with 57.0% of participants having an HIV test for the first time ever pre-operatively (80.5% of participants at MCHC). Self-reported coital activity in the month prior to MMC was 40.4% amongst the participants with more participants from the MCHC reporting sex activity in the last month before VMMC (60.4%; *p*<0.05). Partial protection against HIV and STIs was the main reason for seeking VMMC by most participants at all sites (91.2% of participants), followed by other reasons such as hygiene, cultural, religion, appearance and the perceived enhanced sexual performance after circumcision. Circumcision due to medical indications was the least cited reason for seeking VMMC.

**Table 1:** Baseline age, HIV testing, coital frequency and relationship status of clients volunteering for MMC services

	Site				
		
Characteristic	MCHC (%) n/N	DDH (%) n/N	MDH (%) n/N	Total (%) n/N	*p *
Age group^a^					
12–19	(28.3) 88/311	(21.4) 58/271	(43.8) 7/16	(25.6) 153/598	
20–24	(50.2) 156/311	(40.1) 110/271	(25.0) 4/16	(45.2) 270/598	
25–34	(21.5) 67/311	(32.5) 88/271	(12.5) 2/16	(26.3) 157/598	
35–50	(0) 0/311	(5.5) 15/271	(18.8) 3/16	(3.0) 18/598	
Total	(52.7) 315/598	(45.3) 271/598	(2.7) 16/598		
First ever HIV test	(80.5) 252/313	(24.0) 75/273	(100) 16/16	(57.0) 343/602	<0.05
Sex in the last month	(60.4) 189/313	(17.2) 47/273	(43.8) 7/16	(40.4) 243/602	<0.05
STI treatment in the last three months	(5.6) 18/313	(1.5) 4/273	(12.5) 2/16	(4.0) 24/602	
Relationship status^b^					
SWRP	(62.0) 194/313	(2.9) 8/273	(12.5) 2/16	(33.9) 204/602	
SNRP	(31.6) 99/313	(93.8) 256/273	(62.5) 10/16	(60.6) 365/602	
SNP	(2.9) 9/313	(0.4) 1/273	(12.5) 2/16	(2.0) 12/602	
MSW	(1.0) 3/313	(2.6) 7/273	(6.3) 1/16	(1.8) 11/602	
Other	(1.6) 5/313	(0) 0/273	(0) 0/16	(0.8) 5/602	
Reason for VMMC					
HIV/STI Protection	(95.8) 297/310	(85.3) 233/273	(100) 16/16	(91.2) 546/599	
Medical reasons	(0.3) 1/310	(3.3) 9/273	(0) 0/16	(1.7) 10/599	
Other (culture, religion, hygiene)	(3.9) 12/310	(11.4) 31/273	(0) 0/16	(7.2) 43/599	
Self-report STIs and penile symptoms					
Urethral discharge	(1.0) 3/310	(0.7) 2/272	(6.3) 1/16	(1.0) 6/598	
Genital sores	(0.3) 1/313	(1.1) 3/272	(0) 0/16	(0.7) 4/602	
Erection pain	(0.3) 1/313	(2.6) 7/273	(0) 0/16	(1.3) 8/602	
Swelling of scrotum	(0.3) 1/313	(0) 0/273	(0) 0/16	(1.2) 1/602	
Pain on urination	(0) 0/313	(1.1) 3/273	(0) 0/16	(0.5) 3/602	
HIV negative	(100) 313/313	(100) 273/273	(100) 16/16	(100) 602/602	
Difficulty retracting foreskin	(2.6) 8/313	(5.1) 14/273	(0) 0/16	(3.7) 22/602	

a Four missing age groups

b SRP=single men with regular sexual partners; SNRP=single men with no regular partners; SNP=single men without partners; MSW=married man with single wife.

### Baseline prevalence of co-morbidities and other conditions

Only seven clients (1.2%) presented with at least one co-morbidity other than STIs and penile pathology. Four (0.7%) were on treatment for diabetes, two (0.3%) for bleeding disorders and one (0.2%) for hypertension. All of these participants were circumcised. Data on the prevalence of self-reported STI symptoms and other related conditions/abnormalities are presented in Table 1. Twenty-four participants (4.0%) reported a history of STI treatment in the last three months prior to VMMC. Most participants who reported a history of STI treatment and higher rates of sexual activities in the month prior to VMMC were from the Midlands facilities, (*p*<0.05).

Data on the prevalence of symptoms based on the physical examinations by health care providers are presented in Table 2. There was no association between the reasons for circumcision and the prevalence of STI symptoms or other co-morbidities (*p*=0.183). Phimosis was the most prevalent penile disfigurement and more common in the 12–19 (*n*=12) and the 20–24 (*n*=10) year-old groups (*p*<0.05). There was no correlation between self-reported STIs and penile abnormality symptoms and the findings on clinical examinations of the participants (*κ*=0.062; 95% CI: −0.001–0.144; McNemar's *χ* ^2^=69.450; *p*<0.05).

**Table 2:** Baseline prevalence of penile abnormalities on clinical examination

Penile abnormalities detected		

Condition	*N*	%
Balanitis	0	0.0
Ulcer	6	1.0
Epispadias	0	0.0
Hypospadias	0	0.0
Torsion of the penis	0	0.0
Foreskin attached to glans	0	0.0
Phimosis	26	4.3
Paraphimosis	1	0.2

### Follow-up visits

Most participants (75.6%, *n*=455) were able to return for the 48-hour post-operative visit. The proportion of participants returning for the seven-day visit declined to 51.0% (*n*=307) and 26.1% (*n*=157) by the day 21 visit date. For men aged 20–24 years, the follow-up rates at 2-, 7-, and 21-day was 45.5% (207/455), 46.9% (144/307), and 52.2% (82/157), respectively. In comparison, follow-up rates for men aged 35–49 were 2.4% (11/455) (2 days), 3.6% (11/307) (7 days), and 1.3% (2/157) (21 days). There was no association between the return rates of participants for the follow-up visits and the site of circumcision.

### Post-surgical AEs

Data on the prevalence of AEs observed during the three scheduled visits are presented in Table 3. One intra-surgical complication was observed at MCHC where the participant did not respond to the local anaesthetic and hence VMMC was performed under general anaesthetic. The average rates of moderate and severe AEs were 0.5 and 0.4%, respectively. Three participants (0.4%) were classified as having more than one AE per single visit (included in the AEs reported per visit). The mean period for development of AEs was seven days. The median time to wound healing could not be estimated because the follow-up period was three weeks, and some participants still had incomplete wound healing by that date. However, at 21 days of follow-up, 59.2% (93/157) of participants had complete wound healing.

**Table 3:** Prevalence of adverse events amongst the participants

	Total visits	Moderate AE detected (% recorded visits)	Severe AE reported (% recorded visits)	Two or more AE per visit
2 days	457	(0.7) 3	(0.4) 2	(0.2) 1
7 days	307	(0.3) 1	(0.3) 1	(0.3) 1
21 days	157	(0.6) 1	(0.6) 1	(0.6) 1
Average AE rate		0.5%	0.4%	0.4%

Categorical data on the prevalence of AEs by age group are presented in Table 4. Due to the low numbers of AEs observed, the significance of association between the prevalence of AEs and age groups could not be determined. However, categorical age stratified data shows that older men of 35–56 years had more AEs observed when compared to the younger age groups.

**Table 4:** Adverse events by age group

		48-hour visit		7-day visit		21-day visit		
			
		Moderate	Severe	Moderate	Severe	Moderate	Severe	Average (%)
Age group	12–19	(0.9) 1/115	(0.9) 1/115	(0) 0/79	(0) 0/79	(0) 0/39	(2.6) 1/39	0.7
	20–24	(0.4) 1/207	(0) 0/207	(0) 0/144	(0) 0/144	(1.2) 1/82	(0) 0/82	0.3
	25–34	(0.8) 1/122	(0.8) 1/122	(0) 0/73	(0) 0/73	(0) 0/34	(0) 0/34	0.3
	35–56	(0) 0/11	(0) 0/11	(9.1) 1/11	(9.1) 1/11	(0) 0/2	(0) 0/2	3.0
Total		(0.7) 3/455	(0.4) 2/455	(0.3) 1/307	(0.3) 1/303	(0.6) 1/157	(0.6) 1/157	0.5

Table 5 shows the types of AEs observed at scheduled visits inclusive of mild AEs. Swelling and bleeding were the most frequently observed AEs at 48 hours and 8 (1.3%) participants had wound infections requiring treatment with oral antibiotics. At day 7, wound infection and swelling were the most frequently observed events with 19 (3.2%) participants requiring treatment with oral antibiotics. By the 21st day, wound infection and a few cases of swelling accounted for the majority of AEs observed.

**Table 5:** Types of adverse events detected

	Swelling	Haematoma	Bleeding	Infection	Difficulty urinating	Wound disruption/delayed healing	Problems with appearance	Injury to the glans	
		
	(%) n/N	(%) n/N	(%) n/N	(%) n/N	(%) n/N	(%) n/N	(%) n/N	(%) n/N	Other
48 hour	(28.3) 130/460	(1.3) 6/460	(1.7) 8/460	(1.5) 7/460	(0) 0/460	(0.4) 2/460	(0.9) 4/460	(0) 0/460	2 Cases of stitches coming out due to erection 1 Re-suture 1 Oedema 1 opening of wound during swelling/insufficient skin removed
7 days	(7.2) 22/306	(0) 0/306	(1.0) 3/306	(4.9) 15/306	(0) 0/306	(1.0) 3/306	(0.3) 1/306	(0) 0/306	1 Excess skin removed/debridement 1 Small opening on the frenulum
21 days	(2.5) 4/157	(0) 0/157	(0) 0/157	(7.6) 12/157	(0) 0/157	(1.3) 2/157	(0) 0/157	(0) 0/157	

### Risk factors for AEs

The associations between factors such as the age of participants, the site of presentation, the circumcision procedure and the AE rates could not be inferred in this study due to the low numbers of AEs observed. No significant associations were found between the histories of sexual activity of participants and the reasons for seeking VMMC.

## Discussion

The low AE rates following VMMC in this community are very encouraging; however, client post-surgical follow-up rates remain low. The low AE rates were observed despite the fact that most of the participants came from peri-urban settings where they are prone to conditions of poverty and poor-hygiene due to the high prevalence of informal settlements.

The study confirms the observations made in the randomized controlled trials [4–6] and some that were conducted outside clinical trials that observed AE rates of 1.8% in Orange Farm, SA [14] and 1.2% in Uganda [15]. However, the Orange farm study consisted of more than 14,000 participants in a predominantly circumcising community and the Ugandan study focused on mild AEs. In this study, we found a lower average AE rate for moderate and severe AEs despite the lower numbers of men followed-up. Most of the AEs observed in our study were mild and infrequent, consistent with observations in other studies. There were no differences in AE rates between the three settings. The younger participants aged 12–24 were the most likely to present for VMMC services and had lower rates of AEs. The high demand for VMMC observed amongst young men is encouraging as males of younger age groups are the main target for the VMMC and other HIV prevention strategies in SA [16] given that their HIV prevalence increases substantially from 2.9% in young men aged 15–24 years to 20.9% in men aged 25–49 years [17]. The finding of higher uptake rates of VMMC services and follow-up rates in young men is consistent with other studies, and is attributed to the availability of free time by men in this age group as most are unemployed youth out of school (currently 49% unemployment rate) [18]. Therefore, such youth find time to attend VMMC services compared to the older counterparts. Older men (aged 35–56 years) were most likely to present with AEs than other age groups. However, the small sample of men in these age groups could have influenced this trend. The occurrence of multiple AEs from the same participant was rare.

Except for one participant where the local anaesthetic did not work and who had to undergo VMMC under the general anaesthesia, the in-facility procedures appear to be safe with minimum intra-surgical complications recorded from all study sites. This finding of low intra-surgical complications is consistent with observations from other studies [14].

The predominant symptoms were swelling and mild bleeding that were reported by day 2 of follow-up. The early appearance of symptoms of swelling that is the most frequently observed event at day 7, wherein it was accompanied by appearance of wound infections and is inconsistent with other studies that show bleeding and infection as the most common early stage AEs [19]. Most clients were counselled on maintaining the elevated position of the penis to mitigate the swelling symptoms, as most swelling events were attributed to inappropriate positioning (personal communication with clinicians). There were two severe cases of swelling with resultant opening of the wounds requiring re-suturing. Most swelling symptoms resolved by day 7 following the penis elevation counselling. Two other re-suturing cases were as a result of stitches coming apart due to erections.

The emergence of wound infection within a week of VMMC procedures may indicate some poor wound care practices by some participants over time. More participants appear to start presenting with AEs by day 7 of the follow-up schedule and more AEs were presented during this visit than at any other visit.

However, we found that the prevalence of co-morbidities and self-reported physical abnormalities amongst the participants was low across all three settings. The unreliability of self-reported symptoms with respect to sex and sexually transmitted diseases has been well described [20].

Most participants in this study were young, sexually active, HIV-negative single men without regular sexual partners whose HIV risk is increasing and who stand to benefit the most from VMMC. This low but changing HIV risk is further emphasized by the finding of high prevalence of STIs particularly at the Midlands facilities where the average of STI rate was 6.8%. The Durban site reflected lower STI rates of 1.4% compared to the Ethekwini district rate where it was situated at 0.4% at the time of conducting the study. The national STI prevalence rate was 1.6% (95% CI: 1.5–1.8) in 2011 [21]. The higher rates of STI infection rates in the Midlands could be due to the higher number of rural clients who attend services at its institutions due to the possible higher sexual risk taking by certain rural populations [22] and poor access to condoms and other barrier methods that is prevalent in such settings [23]. The consistent use of condoms was not assessed in this study; therefore it is possible that the lower STI rates may have been influenced by condom use.

Overall the low prevalence of co-morbidities including STIs may have been influenced by the significant number of men who indicated sexual inactivity prior to VMMC. The presentation rates for VMMC by older men who may have been most likely to have co-morbidities associated with chronic lifestyle diseases was also lower and may have contributed to the lower co-morbidity rates. Some participants also came from poor/peri-urban and rural communities where the level of physical activity tends to be higher, e.g. children walking to school, and older men utilizing public transport at points far away from residences and access to fast foods associated with increased risk of chronic lifestyle diseases tends to be restricted. Some authors have attributed such routine physical activities and dietary practices in such communities to the lower prevalence of chronic lifestyle diseases including diabetes and hypertension [24].

The rates of attendance of the follow-up review visits were low in this study. The low rates of follow-up appear to be influenced by the age of the clients and the duration of the follow-up visit. Older men were least likely to return for the review visits and the attendance rates tend to decline over time with fewer men returning after 21 days. This finding is consistent with other studies that attribute the low rates of follow-up among older men to the mostly full-time nature of their employment. In this study, older men who failed to return for review visits had more AEs than younger men who mainly returned for the scheduled visits; a finding common with other studies [25]. It is therefore possible that there could have been more AEs reported if a higher number of older men returned for the follow-up visits.

The site of presentation did not appear to influence the attendance rates as the visit attrition trends were the same at all sites.

Other factors that could influence the high attrition rates of follow-up particularly those observed at 21 days post-surgery include satisfactory wound healing that may have occurred (59.2% presenting with complete wound healing at day 21 (week 3), and socio-economic conditions such as the cost of transport to the clinics. The Kisumu trial [4] showed complete healing in 98.7% of men by day 30 and the Rakai trial [5] up to 88.4% by day 30. However, these two trials did not use stringent follow-up protocol for wound assessment and the timing of observation of wounds classified as either 30 days or four weeks after surgery varied widely. Therefore, the true assessment of proportions healed at specific time points was rendered difficult. Recent studies show up to 70% of HIV negative and 59% of HIV positive men having complete wound healing by week 4 [26]. The negative impact of transport costs on follow-up visits has been shown to be a major factor influencing attendance in other studies [25]. With the VMMC training subsequently having been expanded to the primary health care facilities, some of the clients may have chosen to be reviewed at nearby facilities resulting in poor follow-up the primary VMMC sites.

The study had limitations in that a small percentage of participants particularly in the age groups of 12–14 and those above 50 years were circumcised resulting in fewer review visits of these age groups being conducted. Hence these age groups were included in other age categories. The health care workers who conducted the circumcisions and the follow-up reviews were also well-trained individuals mainly allocated on a full-time basis to provide VMMC services. Therefore the findings may not be adequately generalizable to programmes staffed by providers who are not fully dedicated to circumcision services and with minimal training. There were also cases of client files not fully completed with critical information such as the circumcision method and the participants’ age resulting in the inability to confirm the participants’ eligibility and therefore omission from the study sample. The excluded files may have yielded some critical information on the study outcomes.

## Conclusions

Overall the study confirms that circumcision of men in non-circumcising communities can be undertaken safely. However, there is a need for increased counselling of clients on the importance of adherence to the post-surgical schedules. Our findings indicate the need to formulate guidelines on wound care that considers socio-economic conditions of the communities particularly the older men who are unlikely to return for follow-up visits, but are more likely to present with AEs. An active surveillance system for AEs driven by outreach providers to identify and report AEs may be more desirable than the passive surveillance in use given its limitations on timeliness and data completeness. Health systems strengthening and training of health providers, particularly those involved with the documentation of clinical data and maintenance of records, are required in order to determine the full public health impact of the VMMC programme.
